# The role of S100A9 as a diagnostic and prognostic biomarker in septic shock

**DOI:** 10.1371/journal.pone.0325679

**Published:** 2025-06-06

**Authors:** Yingxue Hu, Qinghui Yang, Xiao Liu, Li Yuan, Fang Yan, Yang Li, Pingwei Ni

**Affiliations:** 1 Department of Respiratory and Critical Care Medicine, North Sichuan Medical College, Nanchong, Sichuan, China; 2 Department of Respiratory and Critical Care Medicine, Chengdu Fifth People’s Hospital, Chengdu, Sichuan, China; 3 Geriatric Diseases Institute of Chengdu, Department of Geriatrics, Chengdu Fifth People’s Hospital, Chengdu, Sichuan, ChinaGeriatric Diseases Institute of Chengdu, Department of Intensive Care Medicine, Chengdu Fifth People’s Hospital, Chengdu, Sichuan, ChinaCenter for Medicine Research and Translation, Chengdu Fifth People’s Hospital, Chengdu, Sichuan, China,; 4 Geriatric Diseases Institute of Chengdu, Department of Clinical Laboratory, Chengdu Fifth People’s Hospital, Chengdu, Sichuan, China; Far Eastern Memorial Hospital, TAIWAN

## Abstract

**Background:**

Sepsis is a severe and potentially fatal systemic condition marked by excessive immune defense against infection. Within this research, we explored the serum concentration of S100A9 during hospital admission, aiming to assess its role and reliability as a viable biomarker for identifying septic shock and predicting mortality risk in sepsis. Furthermore, we explored whether combining S100A9 with conventional assessment tools could enhance diagnostic precision and prognostic accuracy, offering valuable insights to support early intervention and personalized treatment strategies for sepsis.

**Methods:**

This study comprised 575 participants overall, with sepsis patients classified into non-shock and shock groups based on the severity of their condition. Additionally, age- and gender-matched ICU control cohort and healthy control group were recruited to ensure wide applicability and strong comparability of the findings. Enzyme-linked immunosorbent assay utilized for detecting serum S100A9 levels in subjects within 24 hours of admission, ROC curves were used to evaluate the disease identification and prognostic analysis. Differences in survival outcomes among patients with varying levels of S100A9 expression were analyzed using the Kaplan-Meier method.

**Results:**

Serum S100A9 concentrations were elevated in septic patients upon admission. In the diagnosis of patients with septic shock, S100A9 performed similarly to APACHE II, and a considerable enhancement was noted in the sensitivity of detecting septic shock when S100A9 was combined with lactate and APACHE II. At the initiation of ICU stay, the area under the receiver operating characteristic curve (AUC) for the association between serum S100A9 levels and 28-day mortality was 0.78. This value surpassed the AUCs for IL-6 (0.66), procalcitonin (0.60), lactate (0.58) and C-reactive protein (0.47). Furthermore, septic patients with elevated serum S100A9 levels (≥ 630.77 pg/ml) exhibited lower survival rates compared to those with lower concentrations (< 630.77 pg/ml).

**Conclusion:**

S100A9 is a promising biomarker for diagnosing septic shock and forecasting clinical outcomes in patients with sepsis. In addition, S100A9 has good predictive efficacy for the risk of death in sepsis patients.

## Introduction

Sepsis is a lethal systemic condition induced by infection, defined by extensive inflammation, immune system dysregulation, and microcirculatory disruption [1,2]. As a major health problem worldwide [3], the mortality rate of hospitalisation due to sepsis is 25–30%, and hospital mortality for septic shock can even reach an alarming 40–60% [4–6]. Consequently, proactive diagnosis and prognosis assessment of sepsis are crucial to prevent progression and reduce mortality [7,8]. There is no gold standard biomarker that enables monitoring of sepsis diagnosis and disease progression, but the overall heterogeneity of sepsis patients suggests that a combination of biomarkers should be used to comprehensively assess early disease progression and eventual clinical outcomes [9]. Currently, various clinical laboratory markers are frequently employed to evaluate the severity of sepsis and track its progression, including biomarkers like C-reactive protein (CRP) and procalcitonin (PCT) [10–12]. However, these currently widely used biomarkers can be elevated after the occurrence of trauma or surgery, besides being raised in sepsis [13]. Moreover, the sensitivity and specificity of these biomarkers are very limited due to their different release times at the beginning of infection [14]. Therefore, we need to continue to uncover biomarkers that better predict sepsis severity and mortality outcomes and use this as a basis for more rapid patient stratification and thus early intervention in disease prognosis.

The development of sepsis is fundamentally driven by an imbalance throughout the inflammatory pathway [15,16]. The immune system is quickly triggered upon pathogen invasion, with macrophages, a crucial component of innate immunity, releasing significant quantities of pro-inflammatory cytokines [17,18]. However, as part of the development of inflammation, stressed cells are capable of releasing cytoplasmic and nuclear proteins, some of which are involved in signaling to trigger or amplify inflammation. These signaling molecules can be categorized as damage-associated molecular patterns (DAMPs) [19]. The S100 protein family exhibits a high level of structural similarity and remarkable versatility, with at least 25 distinct members of this subgroup have been described [20]. Some of these S100 proteins interact with pattern recognition receptors (PRRs) as DAMPs in the primary immune response to microbial infections and act as modulators of inflammation [21]. S100A9 is an inflammation-associated s100 protein that can be released into the extracellular space in response to cell injury, necrosis, or stress, and acts as one of the DAMPs to activate the immune system [22]. When inflammation occurs, necrotic cells passively release or macrophages actively secrete S100A9, resulting in a significant upregulation of its expression level [23]. High expression levels of S100A9 can directly bind to TLR4 and trigger the NF-κB signaling cascade, which further promotes the secretion of pro-inflammatory cytokines [24]. on the other hand, S100A9 can also bind to RAGE, which leads to oxidative stress and exacerbation of chronic inflammation, such as in atherosclerosis and inflammatory bowel disease [25]. In addition, S100A9 plays a pivotal role in neutrophilic asthma (NA) by enhancing neutrophil activation, NET formation and macrophage polarisation [26]. Based on these cascade activations of inflammatory pathways involved in S100A9, and further constructing a mouse model of sepsis, it can be found that the initial S100A9 level in the sepsis model group was considerably higher than that in the baseline group [27,28]. We therefore hypothesize that S100A9 may be elevated in the early stage of sepsis, and may serve as a potential biomarker for early diagnosis of septic shock. Based on this research background, the aim of this study was to investigate the diagnostic potential of S100A9, and the correlation of its serum levels with disease progression and patient prognosis was investigated.

## Materials and methods

### Ethics approval

The study complied with the ethical standards set forth in the Declaration of Helsinki. The research methodology was assessed and sanctioned by the National Ethics Committee of Chengdu Fifth People’s Hospital (2023-001-01). A total of 575 adult subjects were recruited between January 11, 2023 and January 10, 2024. All subjects granted written informed consent after being informed about the study’s objectives and potential risks, ensuring their participation was both voluntary and well-informed. An authorized agent may make medical decisions on behalf of a comatose patient if the patient is unable to consent on his or her own. To ensure privacy, all gathered data anonymized before conducting the analysis.

### Study design

According to the latest sepsis-3 definition, the diagnosis of sepsis requires the fulfillment of the following two core conditions: first, a confirmed or suspected infection must be present, and second, an increase of ≥2 points in the Sequential Organ Failure Score (SOFA) from the baseline value must be present. For the diagnostic criteria of septic shock, the patient is required to meet the diagnosis of sepsis, as well as the following conditions: 1) Vasoactive medications are still required to maintain mean arterial pressure ≥65 mmHg after adequate rehydration; 2) a sustained blood lactate level >2 mmol/L, and exclusion of any other cause of hypotensive state.

This study enrolled 363 patients who met the criteria used to diagnose sepsis. The outcome of this study was defined as all-cause death at any time point within 28 days from ICU admission, and patients were recorded as “alive” if they survived during the 28-day follow-up period. To ensure data integrity and analytical precision, 28 patients with incomplete or missing critical information were omitted from the analysis. Consequently, an aggregate of 335 patients were included and stratified into two subgroups based on the gravity of their condition: the non-shock sepsis group (n = 141) and the septic shock group (n = 194). In addition, 120 critically ill ICU patients without sepsis (cerebral trauma, intracranial hemorrhage and cerebrovascular accidents) were identified as the comparator group for non-septic ICU patients. Simultaneously, 120 healthy participants were enrolled as the healthy control group.

Inclusion criteria for this study included age ≥ 18 years and a diagnosis of sepsis or septic shock that met Sepsis-3 criteria. All enrolled cases were required to complete S100A9 testing within 24 hours of ICU admission. In addition, the clinical data of the included cases were complete. Those on long-term immunosuppressive drugs or undergoing radiotherapy and pregnant or lactating women were excluded; those involved in other interventional clinical trials and those with incomplete data collection or lost to follow-up were also excluded.

### Clinical data collection

After enrolment, the clinical and demographic data of all participants were documented, including age, sex, coexisting diseases, SOFA score and APACHE II score within the initial 24 hours. Entire study period, additional clinical and microbiologic data were gathered, including microbiologic culture results (such as infection sources and identified pathogens) and clinical outcomes. The clinical parameters recorded included serum lactate, PCT, IL-6, and CRP.

### Sample collection

For those with a definite diagnosis of sepsis, blood was collected within 24 hours of admission, and all of them collected 6 ml of fasting venous blood in the morning, and serum S1009A level was measured by enzyme-linked immunosorbent assay. Lactate, PCT, IL-6, CRP and other indices measured on the same day were recorded at the same time.

### ELISA for serum S100A9 level

After thawing of frozen serum, we used the Human S100A9 ELISA Kit (Protein S100-A9 ELISA Kit, Wuhan Fine Biotech Co,Ltd) to detect the concentration of S100A9 in serum.

### Statistical analysis

Data analysis and plotting were achieved through SPSS version 27.0 and GraphPad Prism version 9.0. Due to non-normal distributions, non-parametric data were expressed as medians with interquartile ranges(25%–75%). For continuous parameters with non-normal distributions, the nonparametric Mann-Whitney test was used for between-group comparisons, and the Krusk-Wallis test was used for multiple group comparisons with Bonferroni correction. Classified data were illustrated as percentages (%) and analyzed using the χ² test for group comparisons. Logistic regression modeling was used to identify independent risk factors for sepsis. To further investigate the role of biomarkers in disease prediction, we assessed their predictive performance by generating ROC curves and calculating the AUC to measure their predictive accuracy. Comparison of differences in ROC curves using the DeLong test. The 28-day survival rate was analyzed using the Kaplan-Meier method, with variations in survival outcomes assessed by the log-rank test. The significance level for statistical analysis was set at P < 0.05, with all P values below this threshold regarded as indicating statistically significant differences.

## Results

### Baseline characteristics

An overall count of 575 participants were incorporated into the derivation cohort. After diagnostic grouping on day 1, baseline data were collected for four distinct patient groups (Table 1). The differences in age and gender among the subgroups were not statistically significant. Among them, the 28-day mortality rate was 48.97% in the septic shock group, significantly higher than the 9.93% observed in the septic non-shock group.

**Table 1:** Baseline characteristics of all subjects on day 1.

	Sepsis (n = 141)	Septic shock (n = 194)	ICU control (n = 120)	Healthy control (n = 120)	P value
Sex [male] (%)	78(55.32%)	123(63.40%)	73(60.83%)	66(55.00%)	0.35
Age, median [Q1-Q3]	70(59-76)	71(65-80)	69(59-78)	70(57-80)	0.20
Comorbidities n (%)					
Hypertension	74 (52.48%)	91 (46.91%)	65 (54.17%)	48 (40.00%)	0.11
Malignancy	11(7.80%)	16(8.25%)	5(4.17%)	2(1.67)	0.06
Diabetes type 2	46(32.62%)	64(32.99%)	38(31.67%)	29(24.17%)	0.28
COPD	20(14.18%)	28(14.43%)	3(2.50%)	2(1.67%)	<0.001
Chronic kidney disease	13(9.22%)	16(8.25%)	4(3.33%)	–	0.11
APACHE II score	19 [16–24]	28 [21–33]	–	–	<0.001
SOFA score on day 1	5 [3–7]	8 [5–11]	–	–	<0.001
Length of ICU stay	7 [5–12]	8 [4–14]	–	–	0.48
Mechanical ventilation percentage	46(32.62%)	138(71.13%)	–	–	<0.001
28-days mortality	14(9.93%)	95(48.97%)	–	–	<0.001
S100A9(pg/ml)	364.43(203.40, 561.56)	747.82(430.63, 1501.63)	280.99(184.11, 457.12)	268.20(134.97, 358.10)	<0.001

Note: Values are expressed as median (interquartile range) or number (percentage). P-value: Kruskal-Wallis test and Bonferroni correctionp; P < 0.05 was considered statistically significant. SOFA Score Sequential Organ Failure Assessment Score, APACHE II score Acute Physiology and Chronic Health Evaluation II score

#### Characteristics of serum S100A9 level fluctuations in sepsis patients.

**Fig 1:**
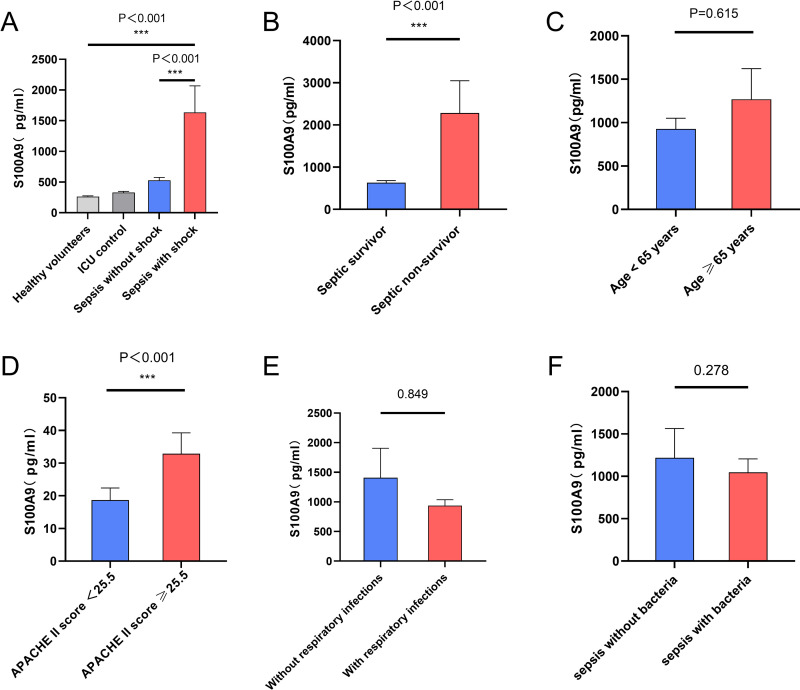
Multidimensional comparison of serum S100A9 levels at admission in different subgroups of septic patients. **(A)** Enzyme-linked immunosorbent assay was used to determine S100A9 levels in serum samples from 194 septic shock patients, 141 sepsis without shock, 120 non-septic ICU patients, and 120 healthy volunteers. **(B)** S100A9 levels in serum from non-survivors and survivors of septic patients. **(C)** Serum S100A9 levels in non-elderly sepsis patients and elderly sepsis patients. **(D)** S100A9 levels in sepsis patients grouped by APACHE II score threshold. **(E)** Serum S100A9 levels in patients with sepsis uncomplicated respiratory infection and in patients with sepsis complicated by respiratory infection. **(F)** Serum S100A9 levels in patients with sepsis uncomplicated bacterial infection and in patients with sepsis complicated by bacterial infection. p ≤ 0.05 was considered statistically significant. * indicates P < 0.05, ** indicates P < 0.01, *** indicates P < 0.001.

Upon ICU admission, individuals diagnosed with septic shock exhibited higher serum S100A9 levels against the septic non-shock patients, and the difference between the two groups was statistically significant (p < 0.001)(Fig 1A). The median concentrations of S100A9 on admission were 364.43 pg/ml (203.40, 561.56) in patients with sepsis, 747.82 pg/ml (430.63, 1501.63) in patients with septic shock, 280.99 pg/ml (184.11, 457.12) in non-septic ICU patients, and 268.20 pg/ml (134.97, 358.10) in healthy controls. Synchronously, the serum S100A9 levels of non-surviving and surviving sepsis patients were analysed, and a statistically significant difference was identified across both groups(Fig 1B).

#### Difference in serum S100A9 levels between elderly and non-elderly patients with sepsis.

Age stratification to assess differences in serum S100A9 levels in nonelderly (<65 years) and elderly (≥65 years) septic patients(Fig 1C). The median concentrations of S100A9 were 486.34 pg/ml (381.08, 1063.16) in the non-elderly patients, 540.65 pg/ml (312.88, 1110.92) in the elderly patients. No significant statistical difference was detected across both groups (P = 0.615). The results may indicate some adaptive or compensatory mechanisms of immune response and inflammatory regulation in elderly patients, and these changes may not significantly affect S100A9 levels.

#### Difference of S100A9 concentrations between sepsis groups according to the APACHE II score.

Patients with sepsis with higher APACHE II scores are associated with poorer prognosis [29]. Sepsis patients were partitioned into two groups based on the APACHE II score threshold: high-risk patients with scores ≥25.5 and low-risk patients with scores <25.5. The analysis revealed that serum S100A9 levels showed significant elevation in patients with scores ≥25.5 compared to those with scores below 25.5(Fig 1D). This suggests that elevated S100A9 levels can be used as a predictor of sepsis exacerbation, which, in combination with the APACHE II score, can provide valuable clinical information about the severity of the disease and prognosis.

#### Difference of S100A9 levels in different infection sites.

Differences in serum biomarker levels at different sites of infection can be used to differentiate between different types of infections, assess the severity of the infection and the prognosis of patients [30]. Analysis of serum S100A9 levels based on infection site upon patient admission revealed no statistically significant differences across various infection sites (P = 0.969) (S1 Table). These findings indicate that serum S100A9 concentrations are unlikely to be substantially influenced by the infection site and are instead more closely linked to the systemic inflammatory response or overall disease severity. Further investigation identified respiratory infections as the most prevalent source of infection in sepsis patients, aligning with previous research [31]. However, there was no statistical difference in S100A9 levels was observed between septic patients with respiratory tract infection and those without(Fig 1E).

#### The difference in S100A9 levels was analyzed based on bacterial culture results.

Among the 335 sepsis patients, 95 cases were verified to have bacterial infections through microbiological culture, while 240 cases showed no evidence of bacterial infection. A more detailed subgroup analysis of microbiological culture results revealed that gram-negative bacteria and mixed infections constituted a significant proportion of cases, indicating they may serve as the primary causative agents of sepsis(S2 Table). However, serum S100A9 levels in patients combined with bacterial infections were not statistically different from those in patients without combined bacterial infections(Fig 1F).

#### Identification of independent risk factors for septic shock.

We used logistic regression modeling to identify independent risk factors for sepsis by analyzing patients’ physiological variables, comorbidities, and traditional laboratory biomarkers. These variables included sex, age, comorbidity with diabetes or hypertension, APACHE II score, S100A9, PCT, lactate, and CRP. Univariate logistic regression analyses showed that age (OR = 1.017, 95% CI 1.000–1.035, P = 0.048), Sex (OR = 1.399, 95% CI (0.899–2.177), P CT, lactate, and CRP. Univariate logistic regression analyses showed that age (OR = 0.137), APACHE II (OR = 1.116, 95% CI 1.097–1.178, P < 0.001), S100A9 (OR = 1.001, 95% CI 1.001–1.002, P < 0.001), Lactate (OR = 1.364, 95% CI 1.197–1.554, P < 0.001), and PCT (OR = 1.013, 95% CI 1.006–1.021, P < 0.001). All significant univariate variables were then subjected to multivariate logistic regression analysis, which demonstrated that APACHE II (OR = 1.116, 95% CI 1.073–1.161, P < 0.001), S100A9 (OR = 1.001, 95% CI 1.000–1.001, P < 0.001) and Lactate (OR = 1.212, 95% CI 1.042–1.409, P = 0.013) were independent risk factors for septic shock(Table 2).

**Table 2:** Logistic regression analyses were performed to analyze the variables associated with the diagnosis of septic shock.

Variable	Univariate analysis		Multivariate analysis	
	OR (95% CI)	P-value	OR (95% CI)	P-value
Sex	1.399(0.899-2.177)	0.137	–	–
Age	1.017(1.000-1.035)	0.048	1.016(0.995-1.038)	0.125
Diabetes type 2	1.017(0.641-1.614)	0.944	–	–
Hypertension	1.250(0.810-1.930)	0.314	–	–
APACHE II	1.116(1.097-1.178)	<0.001	1.116(1.073-1.161)	<0.001
S100A9	1.001(1.001-1.002)	<0.001	1.001(1.000-1.001)	<0.001
Lactate	1.364(1.197-1.554)	<0.001	1.212(1.042-1.409)	0.013
PCT	1.013(1.006-1.021)	<0.001	1.006(0.996-1.015)	0.243
CRP	1.001(0.998-1.003)	0.670	–	–

Note: P < 0.05 means statistically significant.

#### Identifying independent risk factors for septic death outcomes.

To clarify whether S100A9 could be used as an independent risk factor for predicting sepsis death, a one-way regression analysis was first performed, which showed that APACHE II score, S100A9 level, lactate value, PCT, and IL-6 all showed a significant positive correlation with the risk of patient death, and all of them reached the level of statistical significance (P < 0.05). Further multifactorial regression analyses, after controlling for the effects of other variables, conclusively identified APACHE II and S100A9 as independent influences in predicting the risk of death from sepsis, with both showing statistically significant (P < 0.001) (Table 3).

**Table 3:** Logistic regression analysis of variables associated with mortality outcomes in diagnosed sepsis.

Variable	Univariate analysis		Multivariate analysis	
	OR (95% CI)	P-value	OR (95% CI)	P-value
Sex	1.228(0.767-1.967)	0.392	–	–
Age	1.006(0.988-1.025)	0.486	–	–
Diabetes type 2	0.822(0.508-1.331)	0.426	–	–
Hypertension	1.223(0.773-1.933)	0.390	–	–
APACHE II	1.149(1.108-1.191)	<0.001	1.144(1.098-1.193)	<0.001
S100A9	1.001(1.000-1.001)	<0.001	1.001(1.000-1.001)	<0.001
Lactate	1.134(1.030-1.247)	0.010	–	–
PCT	1.008(1.002-1.015)	0.014	–	–
CRP	0.999(0.996-1.001)	0.372	–	–
IL-6	1.359(1.194-1.546)	<0.001	–	–

Note: P < 0.05 means statistically significant.

#### The potential role of S100A9 in diagnosing septic shock.

**Fig 2:**
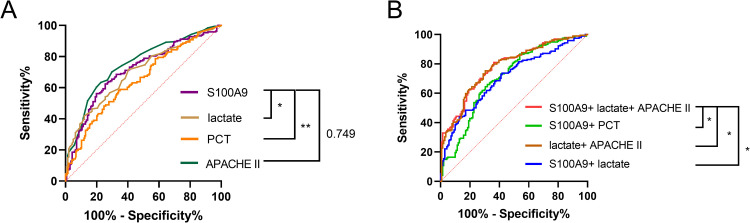
The ROC curves of indicators for the early detection of septic shock. **(A)**The ROC curves of indicators for the detection of septic shock (lactate, PCT, S100A9, APACHE II). **(B)**The predictive values of S100A9+lactate, S100A9+PCT, and S100A9+APACHE II+lactate were calculated by binary logistic regression to calculate ROC curves. ROC curves for the early combined test indices of septic shock (S100A9+lactate, S100A9+PCT, S100A9+APACHE II+lactate). * indicates P < 0.05, ** indicates P < 0.01, *** indicates P < 0.001.

This study evaluates the potential of S100A9 in foreseeing the potential for septic shock by presenting the ROC curve for its diagnostic performance. While identifying patients with septic shock, the analysis revealed that S100A9 (AUC = 0.74, 95% CI 0.68–0.79, P < 0.0001) performed similarly to Apache II but superior to lactate and PCT(Fig 2A, Table 4). The AUC for S100A9 combined with lactate and APACHE II in estimating the risk of septic shock improved from 0.74 (95% CI 0.68–0.79, P < 0.001) to 0.81 (95% CI 0.76–0.86, p < 0.001), and the sensitivity for detecting septic shock was also improved (from 65.50% to 74.20%)(Fig 2B, Table 4).

**Table 4:** The AUC and optimal study parameter thresholds of septic shock patients and their associated diagnostic and efficacy values.

Parameter	AUC (95% CI)	Cutoff Value	SE (%)	SP(%)	PPV (%)	NPV (%)	P-value
APACHE II	0.75(0.70-0.80)	24.5	63.40	77.30	79.35	60.56	<0.001
S100A9(pg/ml)	0.74(0.68-0.79)	539.33	65.50	74.50	77.91	61.05	<0.001
Lactate(mmol/L)	0.66(0.60-0.72)	2.15	67.50	59.60	69.68	57.14	<0.001
PCT(ng/ml)	0.65(0.59-0.71)	3.11	66.00	58.20	68.11	54.67	<0.001
S100A9+Lactate	0.76(0.71-0.82)	–	76.30	67.40	76.17	66.90	<0.001
S100A9+PCT	0.74(0.69-0.80)	–	67.50	73.00	77.51	62.05	<0.001
Lactate+APACHE II	0.78(0.73-0.83)	–	75.30	69.50	77.25	67.12	<0.001
S100A9+APACHE II+Lactate	0.81(0.76-0.86)	–	74.20	74.50	77.01	68.63	<0.001

Note: AUC the area under the ROC curve; Cut-off value the optimal cutoff points for the sample diagnosis of septic shock. P < 0.05 means statistically significant.

#### Predictive value of S100A9 for 28-day mortality in patients with sepsis.

**Fig 3:**
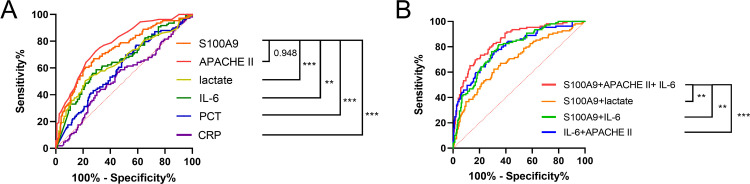
The ROC curves for indicators related to 28-day mortality in sepsis. **(A)** ROC curves of S100A9, APACHE II, IL-6, lactate, PCT, and CRP. **(B)** The ROC curves for the combined test indices of 28-day mortality in patients with sepsis (S100A9+lactate, S100A9+IL-6, S100A9+APACHE II + IL-6). * indicates P < 0.05, ** indicates P < 0.01, *** indicates P < 0.001.

To identify effective methods for projecting 28-day mortality in sepsis patients, we conducted a comprehensive analysis of various clinical scoring systems and admission laboratory markers for their prognostic value in patient survival. The evaluated indicators included S100A9 levels, the APACHE II score, IL-6, lactate, PCT, and CRP. The predictive accuracy of each parameter was systematically assessed through ROC curve analysis and corresponding AUC calculations(Fig 3A, Table 5). Within the six prognostic factors analyzed, the AUC for S100A9 concentration at admission was similar to APACHE II and higher than clinical laboratory indicators such as IL-6, lactate, PCT, and CRP. When S100A9 was combined with APACHE II and IL-6 to predict 28-day mortality in sepsis, the AUC of the combined model increased from 0.78 (95% CI 0.73–0.83, p < 0.001) to 0.84 (95% CI 0.80–0.89, p < 0.001), demonstrating improved predictive performance(Fig 3B, Table 5).

**Table 5:** The AUC and optimal study parameter thresholds for 28-day mortality in patients with sepsis and their associated diagnostic and efficacy values.

Parameter	AUC (95% CI)	Cutoff Value	SE (%)	SP (%)	PPV (%)	NPV (%)	P-value
APACHE II	0.78(0.73-0.83)	25.5	72.50	70.80	54.48	84.21	<0.001
S100A9(pg/ml)	0.78(0.73-0.83)	630.77	72.50	72.10	55.63	84.46	<0.001
Lactate(mmol/L)	0.58(0.51-0.65)	3.55	43.10	78.30	49.48	74.37	0.17
IL-6(pg/ml)	0.66(0.59-0.72)	394.47	58.70	70.40	48.85	77.94	<0.001
PCT(ng/ml)	0.60(0.53-0.66)	2.47	69.70	48.70	35.56	70.97	0.003
CRP(mg/L)	0.47(0.40-0.54)	190.7	30.30	73.00	22.15	78.57	0.343
S100A9+Lactate	0.78(0.73-0.83)	–	69.70	83.20	65.63	80.75	<0.001
S100A9+IL-6	0.79(0.74-0.84)	–	81.70	65.50	53.29	88.10	<0.001
IL-6 + APACHE II	0.79(0.74-0.84)	–	76.10	69.00	71.55	77.23	<0.001
S100A9+APACHE II + IL-6	0.84(0.80-0.89)	–	69.70	83.20	66.67	85.07	<0.001

Note: AUC the area under the ROC curve; Cut-off value the optimal cutoff points for the prediction of 28-day mortality in patients with sepsis. P < 0.05 means statistically significant.

#### Results of the survival curve.

**Fig 4:**
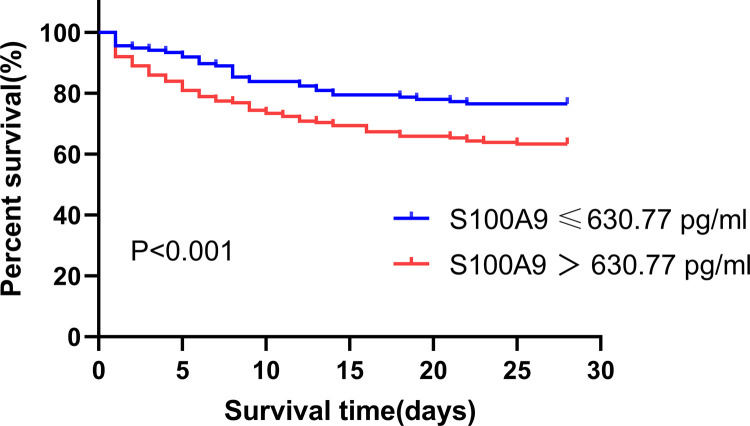
Kaplan–Meier estimator analysis of S100A9 for 28-day survival. The cut off value of S100A9 for the estimated 28-day mortality of patients with sepsis was 630.77 pg/ml.

Derived from the ROC curve analysis, the optimal threshold for predicting 28-day mortality in individuals was identified as 630.77 pg/mL. The results showed that patients with high S100A9 concentrations (≥630.77 pg/mL) had an inferior survival rate than those with low concentrations (<630.77 pg/mL)(P < 0.001)(Fig 4).

## Discussion

Current studies report a short-term mortality rate climbing to 20% among individuals with septic shock [32]. Early diagnosis of sepsis is vital for reducing mortality, but there is currently no gold standard for diagnosis [33]. S100A9 plays a key role in inflammatory responses and immune regulation [34]. Building on this theoretical framework, we assessed the predictive value of S100A9 for disease progression and mortality risk by evaluating its performance against commonly used clinical sepsis-related laboratory markers. First, S100A9 is aligned with the progression and prognosis of sepsis. Serum S100A9 levels were higher within the sepsis cohort on admission in comparison to both control groups. Non-surviving sepsis patients exhibited elevated serum S100A9 concentrations than surviving patients. In addition, we confirmed the predictive value of S100A9 as an independent risk factor for sepsis by multifactorial logistic regression analysis. Univariate logistic regression analysis showed that S100A9 presented a significant correlation with the risk of septic shock development, except for the traditional indicators APACHE II and lactate (P < 0.001). More importantly, multifactorial logistic regression analysis maintained the independent predictive value of S100A9 after correcting for confounders such as age and comorbidities, and this result was also validated in the prediction of mortality risk (P < 0.001). Secondly, S100A9 in combination with APACEH II and lactate improved the sensitivity of septic shock diagnosis. In addition, S100A9 has the same potential in predicting mortality outcomes in septic patients. Furthermore, survival curve analysis highlighted that sepsis patients with high serum S100A9 levels at admission had a lower survival rate.

Excessive inflammation and immunosuppression caused by sepsis usually leads to tissue damage [35,36]. S100A9 is significantly upregulated in infections, metabolic inflammation, immune system dysfunction and degenerative diseases [37]. S100A9 is significantly upregulated in infections, metabolic inflammation, immune system dysfunction and degenerative diseases [38]. In numerous studies related to sepsis, S100A9 plays an active pro-inflammatory role. After CLP was used to treat mice, the amount of S100A9 in plasma and lung tissue climbed significantly, causing the formation of pulmonary edema and tissue damage in septic mice by enhancing the expression of Mac-1 and the production of CXC chemokines [39]. In addition, S100A9 can promote epithelial apoptosis driven by LPS through the IL-17–NFκB–caspase-3 signaling pathway [40]. In sepsis-mediated acute liver injury(ALI), S100A9 promotes inflammation by modulating mitochondrial energy metabolism through the AKT-AMPK signaling pathway [41]. The discovery of these pro-inflammatory mechanisms may be able to reasonably explain the increased serum S100A9 concentrations among sepsis patients.

Early risk assessment plays a pivotal role in managing sepsis patients, with the APACHE II score widely regarded as the benchmark for evaluating risk in critically ill individuals [42–43]. In our study, patients with ascending APACHE II scores on admission had similarly significantly raised serum S100A9 concentrations. However, the APACHE II scoring system has notable limitations that can sometimes yield misleading results. For example, young patients with severe sepsis rarely have comorbid chronic organ failure, and despite the risk of adverse outcomes in these patients, final APACHE II scores may be relatively low [29]. Numerous previous research has revealed that the measurement of blood lactate concentration can provide a reliable marker for assessing altered tissue perfusion, disease severity, and prognosis in septic shock [44–46]. To investigate the potential diagnostic value of S100A9 for septic shock, we found that the AUC of S100A9 was superior to lactate and its specificity was higher by ROC analysis. Current research indicates that measuring multiple biomarkers can mitigate the limitations of relying on a single biomarker, enhancing diagnostic and prognostic accuracy. Furthermore, integrating biomarker data with clinical information has shown promise in effectively stratifying the risk of patients with sepsis, thereby improving personalized treatment approaches [47]. In conclusion, S100A9, as an early inflammatory marker, has better predictive efficacy among common biomarkers associated with sepsis prognosis. Elevated serum lactate levels, which are similar to S100A9, are usually associated with inadequate tissue perfusion and metabolic disturbances in the internal environment. The APACHE II score, although capable of assessing the condition based on a more comprehensive clinical picture, has limitations such as subjective factors. Compared to using lactate or APACHE II alone to predict disease severity, the combined use of clinical parameters and multiple biomarkers significantly improved diagnostic efficacy for diagnosing septic shock.

Several biomarkers have been labeled as important predictors of prognosis in sepsis [48,49]. Serum lactate has been shown to be a valuable prognostic indicator of hypoperfusion [50,51]. While CRP and PCT are widely used to assist in identifying sepsis, numerous studies have reported that their diagnostic and prognostic value is limited [52,53]. Therefore, we conducted a ROC analysis using clinical and laboratory parameters to identify the optimal biomarker or biomarker combination for predicting the prognosis of sepsis patients. Furthermore, S100A9 is more sensitive than lactate and IL-6 for assessing mortality risk in sepsis, while its specificity is basically equivalent to that of lactate and IL-6. As mentioned before, APACHE II has certain limitations. In addition, calculating the APACHE II score upon ICU admission requires numerous clinical parameters, and the variability in criteria among assessors can significantly influence the results [54]. In our study, we combined S100A9 with other multimarkers to predict 28-day mortality in sepsis. The findings revealed that the combination of S100A9 with APACHE II and IL-6 significantly enhanced the ability to predict sepsis-related mortality. These outcomes imply that S100A9 functions as a potentially valuable biomarker for diagnosing septic shock and assessing the probability of death from sepsis.

Despite these findings, there are still many limitations. There are limitations in the clinical applicability of the APACHE II scoring system, and we plan to systematically collect and screen the combinations of parameters with the most predictive value in subsequent studies, and ultimately establish a simplified risk assessment model based on S100A9 to enhance clinical utility. Our study only included adult sepsis patients, and further assessment of children with sepsis was lacking. In addition, this was a single-centre study, so generalisability may be limited, and further studies in multiple centres are needed. Due to the significant clinical heterogeneity of sepsis patients (including disease progression, treatment differences, etc.), the correlation between dynamic serum S100A9 levels and length of hospitalization was not systematically analyzed in this study. These limitations do not affect the prognostic value of S100A9 in this study, but suggest that its clinical translation requires systematic follow-up validation, such as whether dynamic changes in S100A9 provide better predictive efficacy than a single assay, and whether risk stratification of S100A9 can improve therapeutic decision-making, which will need to be confirmed in the future by further prospective studies.

## Conclusion

S100A9 shows great potential as an indicator for diagnosing septic shock and predicting 28-day mortality outcomes in patients with sepsis. Patients with high S100A9 levels have a poorer prognosis. S100A9 in combination with APACHE II and lactate improves the diagnostic efficacy of septic shock. Furthermore, S100A9 in combination with APACHE II and IL-6 enhances the prediction of mortality risk in septic patients.

## Supporting information

S1 Table The S100A9 levels in different infection sites in patients with sepsis. (DOCX)

S2 Table The Pathogens responsible for infections in the patients with sepsis at admission in the derivation cohort. (DOCX)

S3 Table Sequential Organ Failure Assessment (SOFA) Score. (DOCX)

S4 File Original Data. (XLSX)
