# Supplementary material for: The role of S100A9 as a diagnostic and prognostic biomarker in septic shock
Source: PLoS One. 2025 Jun 6;20(6):e0325679. doi: 10.1371/journal.pone.0325679 (PMC12143512; doi:10.1371/journal.pone.0325679)
Supplement: S1 Table — (DOCX) [file pone.0325679.s001.docx]

**S1 Table.** The S100A9 levels in different infection sites in patients with sepsis

| site of infection | Number | Non-survival number |
| --- | --- | --- |
| respiratory tract infection | 169 | 65 |
| urinary tract infection | 39 | 8 |
| intestinal infection | 38 | 11 |
| peritonitis | 39 | 15 |
| biliary tract infection | 28 | 6 |
| skin infection | 11 | 2 |
| liver abscess | 5 | 0 |
| head infection | 4 | 2 |
| endometrial infection | 1 | 0 |
| endocarditis | 1 | 0 |
