# Supplementary material for: The role of S100A9 as a diagnostic and prognostic biomarker in septic shock
Source: PLoS One. 2025 Jun 6;20(6):e0325679. doi: 10.1371/journal.pone.0325679 (PMC12143512; doi:10.1371/journal.pone.0325679)
Supplement: S2 Table — (DOCX) [file pone.0325679.s002.docx]

**S2 Table.** The Pathogens responsible for infections in the patients with sepsis at admission in the derivation cohort

| Etiology of infection | Number | Non-survival number |
| --- | --- | --- |
| Gram-positive bacteria | 13 | 4 |
| Gram-negative bacteria | 44 | 16 |
| Fungus | 8 | 3 |
| Mixed bacteria | 30 | 13 |
