# Supplementary material for: The role of S100A9 as a diagnostic and prognostic biomarker in septic shock
Source: PLoS One. 2025 Jun 6;20(6):e0325679. doi: 10.1371/journal.pone.0325679 (PMC12143512; doi:10.1371/journal.pone.0325679)
Supplement: S3 Table — (DOCX) [file pone.0325679.s003.docx]

**S3 Table.** Sequential Organ Failure Assessment (SOFA) Score

| System | Parameter | 0 | 1 | 2 | 3 | 4 |
| --- | --- | --- | --- | --- | --- | --- |
| Respiration | PaO₂/FiO₂ (mmHg) | ≥400 | <400 | <300 | <200 (with ventilation) | <100 (with ventilation) |
| Coagulation | Platelets (×10³/μL) | ≥150 | <150 | <100 | <50 | <20 |
| Liver | Bilirubin (mg/dL) | <1.2 | 1.2–1.9 | 2.0–5.9 | 6.0–11.9 | ≥12.0 |
| Cardiovascular | BP or Vasopressors | MAP≥70 mmHg | MAP<70 mmHg | Dopamine≤5 or dobutamine (any dose) | Dopamine>5 or norepinephrine≤0.1 | Dopamine>15 or norepinephrine>0.1 |
| CNS | Glasgow Coma Scale | 15 | 13–14 | 10–12 | 6–9 | <6 |
| Renal | Creatinine (mg/dL) or Urine Output | <1.2 | 1.2–1.9 | 2.0–3.4 | 3.5–4.9 or UO<500 mL/day | ≥5.0 or UO<200mL/day |
